# Effect of 3 min whole-body and lower limb cold water immersion on subsequent performance of agility, sprint, and intermittent endurance exercise

**DOI:** 10.3389/fphys.2022.981773

**Published:** 2022-10-10

**Authors:** Wei Zhang, Shoupeng Ren, Xinyan Zheng

**Affiliations:** ^1^ Department of Sports Training, Shenyang Sport University, Shengyang, China; ^2^ Shanghai Frontiers Science Research Base of Exercise and Metabolic Health, Shanghai University of Sport, Shanghai, China

**Keywords:** cold-water immersion, exercise performance, thermoregulation, heat, team-sport

## Abstract

The aim of this study was to investigate the effects of whole-body cold-water immersion (WCWI) and lower-limb cold-water immersion (LCWI) employed during a 15-min recovery period on the subsequent exercise performance as well as to determine the physiological and perceptual parameters in the heat (39°C). Eleven males performed team-sports-specific tests outdoors. The exercise program consisted of two identical exercise protocols (1 and 2) separated by a 15-min recovery period. The participants completed the same tests in each exercise protocol, in the following order: agility *t* test (*t*-test), 20-m sprint test (20M-ST), and Yo-Yo Intermittent Endurance Test Level 1 (Yo-Yo). During the recovery period, a 3-min recovery intervention of a passively seated rest (control, CON), WCWI, or LCWI was performed. The *t*-test and 20M-ST for the CON group were significantly longer during exercise protocol 2, but they were not significantly different between the two exercise protocols for the WCWI and LCWI groups. The completed Yo-Yo distance for the CON and LCWI groups was shorter during exercise protocol 2, but it was not significantly different between the two exercise protocols for the WCWI group. The chest temperature (T_chest_), upper arm temperature (T_arm_), thigh temperature (T_thigh_), mean skin temperature (T_skin_), and thermal sensation (TS) values were lower for the WCWI group than for the CON group; but only the T_thigh_, T_skin_, and TS values were lower for the LCWI group compared to the CON group. The T_chest_, T_arm_, T_skin_, and TS values after the intervention were lower for the WCWI group than for the LCWI group. None of the three intervention conditions affected the core temperature (T_core_), heart rate (HR), or rating of perceived exertion (RPE). These results suggest that WCWI at 15°C for 3 min during the 15-min recovery period attenuates the impairment of agility, sprint, and intermittent-endurance performance during exercise protocol 2, but LCWI only ameliorates the reduction of agility and sprint performance. Furthermore, the ergogenic effects of WCWI and LCWI in the heat are due, at least in part, to a decrease of the T_skin_ and improvement of perceived strain.

## 1 Introduction

Many team sporting events are performed in the heat. Compared to the normothermic ambient conditions, when exercising in the heat, it has been shown that the observed larger increases in core body temperature induce additional cardiovascular and metabolic strain and thermal perceptual load, leading to a reduction in exercise performance, especially in the second half of the match (Mohr et al., 2004). In order to improve the subsequent performance of athletes participating in team sports in the heat, it is particularly important to find a practical body-cooling strategy that can be used during half-time to decrease the body temperature and improve the exercise performance of the athletes.

Cold-water immersion (CWI) is a physical cooling method that consists of immersing the body partially or completely in cold water. Studies have shown that CWI employed both, before (i.e., precooling the body) or in between exercise bouts, can improve the subsequent endurance exercise capacity, muscle strength, and intermittent-sprint performance (Maia-Lima et al., 2017; Boujezza et al., 2018; Egaña et al., 2019). Furthermore, CWI post exercise can promote recovery leading to a better sprint performance after 24 h (Leeder et al., 2019). The beneficial effects of CWI may be related to lowering the body temperature, improving the body’s heat storage capacity, promoting peripheral blood reflux, stimulating parasympathetic nerves, and inhibiting the diffusion of inflammatory factors (Maia-Lima et al., 2017).

Several investigations have shown that whole-body cold-water immersion (WCWI) for 12–15 min can effectively improve cardiovascular function and the subsequent immediate exercise performance after the intervention (Yeargin et al., 2006; Vaile et al., 2008; Boujezza et al., 2018; Leeder, et al., 2019). However, the actual duration of WCWI in these studies was not applicable to half-time breaks of team sports, typically lasting ∼15 min (i.e., soccer, rugby sevens). In this context, Peiffer et al. (2010) reported that post-exercise 5 min of WCWI at 14°C employed within a 15 min recovery period significantly lowered the rectal temperature and maintained the subsequent 4 km cycling time trial performance in the heat. More recently, Egaña et al. (2019) showed that WCWI at 8°C for 2.5 min carried out within a 15 min recovery period maintained the intermittent-sprint capacity at an ambient normothermic temperature. Of note, a high ambient temperature can cause heat stress, further impairing the subsequent exercise capacity of athletes competing in the second half of a team sport. Nevertheless, whether a relatively short period of WCWI (<5 min) can improve the subsequent exercise capacity of athletes in the heat remains to be explored.

Moreover, lower-limb cold-water immersion (LCWI), which submerges the lower limbs no more than the iliac crest, is also a common cooling intervention. Previous studies have shown that LCWI before exercise can enhance the intermittent-sprint performance of athletes in the heat (Castle et al., 2006; Castle et al., 2011; Minett et al., 2011). Collectively, these results indirectly suggest that WCWI and/or LCWI during half-time may improve the performance of athletes during the second half of team sports. However, to the best of our knowledge, no previous study has examined the effects of a relatively short (e.g., <5 min) LCWI or WCWI employed within a 15 min recovery period applicable to half-time breaks of team-sports, on the subsequent intermittent endurance and agility performance of athletes, and whether LCWI achieves similar ergogenic effects to WCWI.

For successful performance in team sports, athletes require not only intermittent capacity and sprint performance but also change-of-direction ability at various times throughout the match (Karakoç et al., 2012; Pojskic et al., 2018). Therefore, the aim of this study was to investigate the effects of WCWI and LCWI within a 15-min recovery period on the subsequent performance of agility, sprint, and intermittent-endurance exercises as well as to determine their thermoregulatory responses, heart rate (HR), thermal sensation (TS), and rating of perceived exertion (RPE) in the heat. We hypothesized that both WCWI and LCWI would attenuate the impairment of the subsequent performance of agility, sprint, and intermittent-endurance exercises of athletes after the cryotherapeutic interventions.

## 2 Materials and methods

### 2.1 Participants

Eleven non-heat acclimated male collegiate soccer players (age: 21.4 ± 2.0 years old; height: 178.3 ± 4.8 cm; weight: 69.9 ± 5.5 kg) volunteered to take part in this study, but they were not accustomed to exercise in the heat at the time of the study. The sample size used was based on a G*Power 3.1 software calculation (*p* = 80% at *α* = 0.05; ES = 0.4; Faul et al., 2007), and eleven participants were sufficient to minimize the probability of type II error for all the variables. The participants were deemed eligible for this study if they met the following criteria: 1) aged between 18 and 23 years old; 2) nonsmoker; 3) had prior soccer experience of at least 2 years; 4) exercised at least three times/week; 5) no history of cardiovascular disease or contraindications such as cold allergy and abrasions; 6) no injury or surgery in the past 6 months. The participants were informed of the procedures, requirements, and possible risks of the experiment prior to signing the informed consent form. The procedures were approved by the Ethics Committee of the university (102772021RT149).

### 2.2 Outdoor environmental conditions

All sessions were carried out at an outdoor playground. This study took place in July 2021 in Yangpu, Shanghai, China (latitude: 31° 27′ N, longitude: 121° 52’ E). The weather forecast was based on an online meteorological service (www.weather.com.cn) to ensure that the experiments took place under consistent outdoor conditions. A portable climate monitoring device was used to measure the wet bulb globe temperature (WBGT; 213A, Kyoto Electronics Manufacturing Co., Ltd., Japan) at the beginning and at the end of each experiment. The WBGT device was held by one hand and kept at a distance of about 1.5 m from the ground. The WBGT was recorded at least 2 min after the device was turned on.

### 2.3 Experimental design

Each participant completed a familiarization trial and three formal trials in a randomized controlled crossover design. In order to avoid potential confusion, the randomization was performed in one block of 11 participants using the platform randomization.com (http://www.jerrydallal.com/random/randomize.htm). The randomization was prepared before the formal experiment. During the familiarization trial, the researchers and the participants were acquainted with all procedures to be employed during the control (CON) condition in order for the participants to become familiar with the equipment and the exercise protocol. The participants completed the two exercise protocols and randomly received one of the following 3-min recovery interventions during intermission in the heat: passively seated rest at ambient temperature (CON), WCWI, and LCWI. All exercise protocols were performed on a standard outdoor track. The track was made of natural rubber and artificial rubber, which was continuously calendared with mixed mineral fillers, stabilizers, and color materials, and was synthesized by high-temperature sulfur hardening at 280–300°C, mechanical embossing, and a double-layer structure. Each experimental condition was separated by at least 5 days and performed at the same time of day to eradicate any influence of circadian rhythm. The participants were required to refrain from strenuous exercise for 48 h and from caffeine or alcohol consumption for 24 h prior to the trials. In addition, the same dietary intake was recorded and maintained for 24 h before the formal experiments, and the same diet was guaranteed for the three experimental visits.

### 2.4 Exercise protocol

**FIGURE 1:**
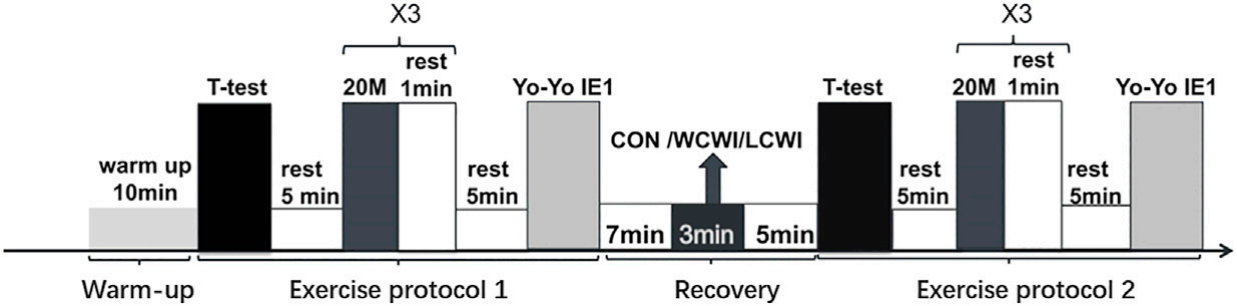
Exercise protocol. Yo-Yo IE 1, Yo-Yo intermittent endurance test level 1. CON, control condition. LCWI, lower-limb cold-water immersion condition. WCWL, whole-body cold-water immersion condition. 1st, exercise protocol 1. 2nd, exercise protocol 2. × 3, repeated three times.

The participants were asked to attach the measuring equipment in advance and sit still at the outdoor track for 20 min. The exercise program consisted of a 10-min warm-up (5 min of jogging and 5 min of stretching) and two identical test exercise protocols separated by a 15-min recovery interval. During the warm up, participants initially jogged for 5 min around the 400-m running track at a self-selected pace, and participants then stretched their neck, arm, and leg muscles for 5 min. Each exercise protocol consisted of three exercise tests in the following order: the agility *t*-test (*t*-test), 20-m sprint test (20M-ST), and Yo-Yo Intermittent Endurance Test Level 1 (Yo-Yo), with a 5-min passive recovery period separating each test (Figure 1). For all conditions, the participants were allowed to consume up to 500 ml of ambient-temperature sports beverage (POCARI SWEAT; Otsuka Pharmaceutical Co., Ltd., Japan) during the warm-up period and the recovery period, and all participants drank all of the sports beverage.

### 2.5 Intervention

Three interventions were used, including passive rest (CON), WCWI, and LCWI. At the end of exercise protocol 1, the participants immediately took off their underwear and training shirt, and then they changed into swimming trunks in the mobile changing shed. The participants received the CON, WCWI, or LCWI intervention randomly at 8–10 min after the start of the intermission. During the CON condition, the participants were passively seated for 3 min in the heat. During the WCWI, the participants were submerged in an inflatable pool with their neck and head out of the water. During the LCWI, the participants sat in an inflatable pool with their legs straight, and the water level was placed at the hip. The water temperature for WCWI and LCWI was maintained at 15°C by a specially designed water refrigeration unit (iCool Portacovery, Gold Coast, Australia). The water temperature selected for this study was in accordance with Vaile et al. (2008) and Peiffer et al. (2010).

### 2.6 Measurements

#### 2.6.1 Exercise capacity

Athletes participating in team sports typically complete a battery of fitness and performance tests to assess their performance. The soccer-specific tests include the *t*-test, which is used to test the agility (Munro and Herrington, 2011); the 20M-ST, which is used to measure speed (Turner et al., 2011); and Yo-Yo, which is used to assess intermittent-exercise performance (Karakoç et al., 2012). These tests have been shown to be reliable, with typical coefficient of variation values for the *t*-test, 20M-ST, and Yo-Yo of 2.9% (Hermassi et al., 2020), 2.7% (Hulse et al., 2013), and 10.2% (Grgic et al., 2019), respectively.

The *t*-test is used to assess the ability of acceleration, deceleration, and lateral movement (Munro and Herrington, 2011). Four cones were arranged in a T shape, with a cone placed 9.14 m from the starting cone, and two further cones placed 4.57 m on either side of the second cone. The participants were asked to sprint forwards 9.14 m from the start line to the first cone, shuffle 4.57 m left to the second cone, then shuffle 9.14 m to the right to the third cone, and shuffle 4.57 m back left to the middle cone before finally back pedaling to the start line. A hand-held stopwatch was used to measure the participant’s time to complete the test to the nearest 0.01 s (300LAP/SPLIT, Seiko, Japan).

The 20M-ST is used to measure acceleration (Turner et al., 2011). The participants performed the 20M-ST three times, and the mean time was recorded as the final test result. A 1-min recovery was given between each trial. A hand-held stopwatch was used to take the participant’s time to complete the test to the nearest 0.01 s (300LAP/SPLIT, Seiko, Japan).

The Yo-Yo intermittent tests are popular for soccer players, and several studies have analyzed their reliability and validity for athletes participating in team sports. They are divided into two commonly used forms: the Yo-Yo intermittent recovery test and the Yo-Yo intermittent endurance test (Grgic et al., 2019). It has been reported that the Yo-Yo intermittent endurance level 1 is a highly reliable tool for determining an individual’s ability to perform intermittent exercise (Karakoç et al., 2012; Póvoas et al., 2016a, Póvoas et al., 2016b). The Yo-Yo intermittent endurance level 1 required two shuttle runs of 20 m that gradually increased in speed as dictated by audio signals. Each run was separated by a 5-s active recovery period, when the participants jogged around a cone positioned 2.5 m behind the start line. Two consecutive failures to reach the finish line before the audio signal indicated test cessation, and the distance covered at that point was the final test result.

#### 2.6.2 Physiological indexes

In this study, the core temperature (T_core_), skin temperature, and HR of the participants were measured. As an index of T_core_, the gastrointestinal temperature was measured *via* an ingestible temperature-sensor capsule (BODYCAP P022-P; BODYCAP S.A.S., France) and was recorded by a telemetric data receiver (BODYCAP e-Viewer Performance; BODYCAP S.A.S., France). Each participant ingested the sensor with water at 2 h before testing. The skin temperature was measured by a wireless thermistor probe (BODYCAP e-Tact; BODYCAP S.A.S., France). The thermistor probe was attached with two Tegaderm bandages to the skin surface of the upper arm (T_arm_), chest (T_chest_), and thigh (T_thigh_). The recorded data of the skin temperature was monitored by the PC/Mac software (eTact watcher, BODYCAP S.A.S., France). The mean skin temperature (T_skin_) was calculated using the formula developed by Roberts et al. (1977): 0.43 × T_chest_ + 0.25 × T_arm_ + 0.32 × T_thigh_. The HR was measured by a HR monitor (model RS400; Polar Electro Oy, Kemple, Finland). T_core_, T_skin_, T_arm_, T_chest_, T_thigh_, and HR were recorded continuously every 3 min during the experiment.

#### 2.6.3 Perceptual indexes

TS (9-point scale ranging from 0 = “very cold” to 8 = “very hot”; Olesen and Brager, 2004) were recorded after the *t*-test and 20M-ST, before and after the Yo-Yo, and every minute during the 3-min intervention. Meanwhile, RPE (5-point scale; Borg, 1982) was documented immediately after the *t*-test and 20M-ST as well as before and after the Yo-Yo.

### 2.7 Statistical analyses

All data were analyzed using SPSS 23.00 software. Data collected was tested for normal distribution using the Kolmogorov-Smirnov Test. Non-normal distributed data were competed with statistical disposal after a log-normal transformation. Ambient temperature under different experimental conditions were analyzed using One-Way Analysis of Variance (ANOVA). Multivariate analysis of variance of repeated measurements (condition × time) were used to analyze the changes of *t*-test and 20M-ST duration, Yo-Yo distance, T_core_, T_skin_, T_arm_, T_chest_ T_thigh_, HR, TS, and RPE. The Greenhouse–Geisser epsilon were used to correct the results when the violation of sphericity prior to analysis test occurred. When interaction effects were identified, post-hoc analyses were carried out with Bonferroni correction. For analysis of variance (ANOVA), partial eta2 (Pη2) was used as a measure of the effect size. The criteria to interpret the magnitude of the effect size were as follows: small, Pη2 = 0.01; medium, Pη2 = 0.06; and large, Pη2 = 0.14 (Cohen, 1992). All data are reported as mean ± standard deviation (SD). Significance was defined as *p* < 0.05.

## 3 Results

### 3.1 Environmental conditions

The WBGT was not significantly different among the three interventions [F (2) = 0.059, Pη2 = 0.003, *p* > 0.05]. The WBGT values when the participants underwent the three interventions were as follows: CON, 39.8 ± 1.2°C; WCWI, 39.7 ± 0.9°C; and LCWI, 39.6 ± 1.1°C.

### 3.2 Exercise capacity

**FIGURE 2:**
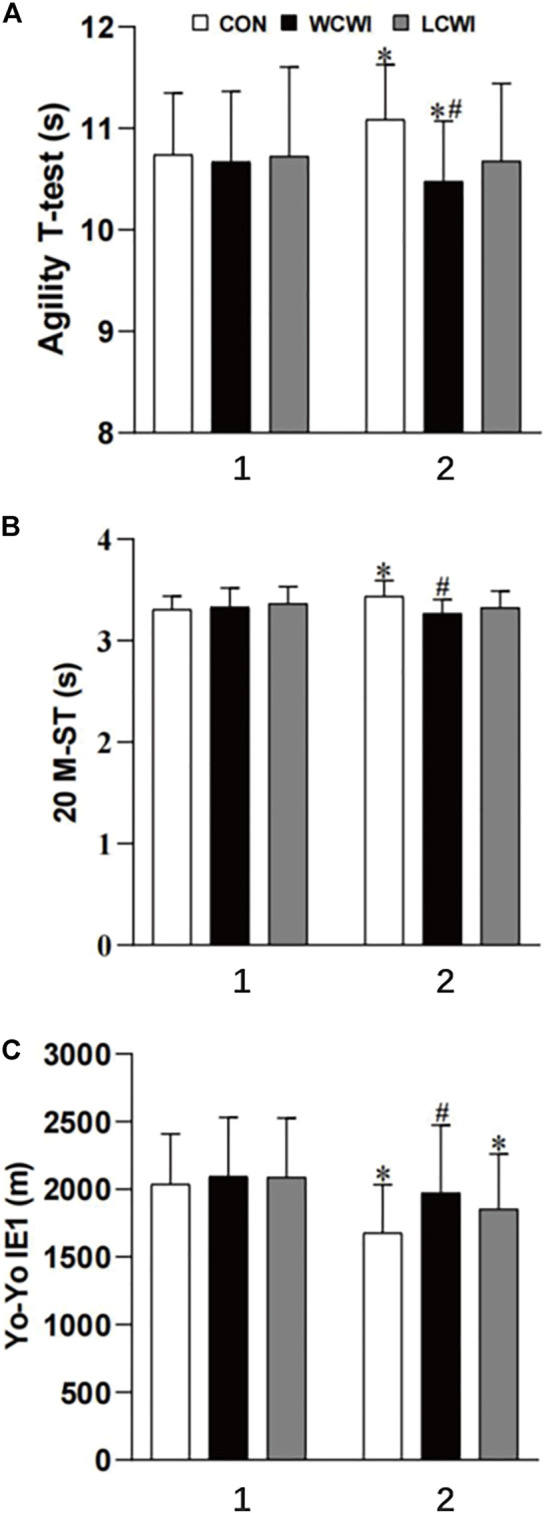
Change in Agility *t*-test **(A)**, 20 m sprint test (20 M-ST) **(B)** and Yo-Yo intermittent endurance test level 1 (Yo-Yo IE 1) **(C)** for control (CON), lower-limb cold-water immersion condition (LCWI) and whole-body cold-water immersion condition (WCWI) conditions. 1, exercise protocol 1. 2, exercise protocol 2. *, significantly different with the exercise protocol 1. #, significantly differently with CON in the exercise protocol 2. The values are shown as mean ± SD. *p* < 0.05 was considered statistically significant.

For the duration of the *t*-test, there was a significant interaction [F (2, 20) = 8.47, *p* < 0.05, Pη2 = 0.46, Figure 2A]. For the CON intervention, the *t*-test was significantly longer in exercise protocol two than in exercise protocol 1 (exercise protocol 1: 10.7 ± 0.6 s; exercise protocol 2: 11.1 ± 0.5 s; *p* < 0.05). In contrast, under the WCWI condition, the *t*-test was significantly shorter during exercise protocol two than during exercise protocol 1 (exercise protocol 1: 10.7 ± 0.7 s; exercise protocol 2: 10.5 ± 0.6 s; *p* < 0.05). There was no significant difference of the *t*-test results between the two exercise protocols under the LCWI condition. During exercise protocol 2, the *t*-test was shorter with the WCWI intervention than with the CON intervention (CON: 11.1 ± 0.5 s, WCWI: 10.5 ± 0.6 s, *p* < 0.05).

For the 20M-ST, there was a significant interaction [F (2, 20) = 13.6, *p* < 0.05, Pη2 = 0.6, Figure 2B]. For the CON intervention, the 20M-ST was significantly longer during exercise protocol two than exercise protocol 1 (exercise protocol 1: 3.3 ± 0.1 s; exercise protocol 2: 3.4 ± 0.2 s; *p* < 0.05). In contrast, under the WCWI and LCWI conditions, there was no significant difference of the 20M-ST results between the two exercise protocols (WCWI, exercise protocol 1: 3.3 ± 0.2 s, exercise protocol 2: 3.3 ± 0.1 s, *p* > 0.05; LCWI, exercise protocol 1: 3.4 ± 0.2 s, exercise protocol 2: 3.3 ± 0.2 s, *p* > 0.05). During exercise protocol 2, the 20M-ST for the WCWI intervention was significantly shorter than that for the CON intervention (CON: 3.4 ± 0.2 s; WCWI: 3.3 ± 0.1 s; *p* < 0.05).

For the completed distances of Yo-Yo, there was a significant interaction [F (2, 20) = 4.13, *p* < 0.05, Pη2 = 0.29, Figure 2C]. With the CON and LCWI interventions, the Yo-Yo distance during exercise protocol two was significantly shorter compared with that of exercise protocol 1 (CON, exercise protocol 1: 2038.8 ± 371.1 m, exercise protocol 2: 1679.5 ± 354.7 m, *p* < 0.05; LCWI, exercise protocol 1: 2088.1 ± 439.0 m, exercise protocol 2: 1853.7 ± 407.2 m, *p* < 0.05). During exercise protocol 2, the completed Yo-Yo distance for the WCWI intervention was significantly longer than that for the CON intervention (CON: 1679.5 ± 354.7 m, WCWI: 1974.6 ± 499.7 m, *p* < 0.05).

### 3.3 Physiological indices

**FIGURE 3:**
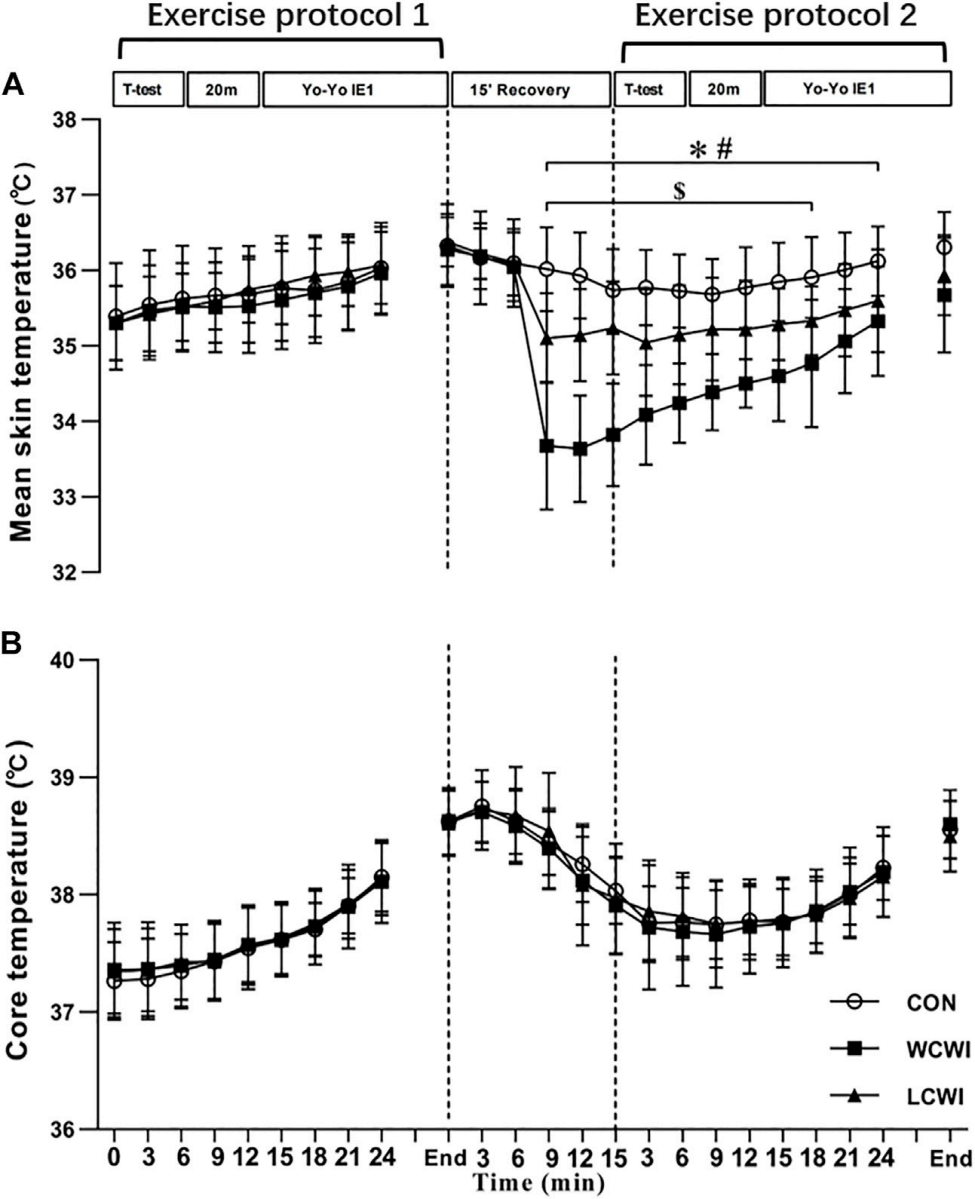
Core temperature **(A)** and mean skin temperature **(B)** changes at different time points during exercise protocol for control (CON), lower-limb cold-water immersion condition (LCWI) and whole-body cold-water immersion condition (WCWI) conditions. *t*-test, agility *t*-test. 20 m, 20 m sprint test. Yo-Yo IE1, Yo-Yo intermittent endurance test level 1. *, CON vs. WCWI. #, CON vs. LCWI. $, WCWI vs. LCWI. The values are shown as mean ± SD. *p* < 0.05 was considered statistically significant.

For T_skin_, there was a significant interaction effects [F (46, 460) = 17.18, *p* < 0.05, Pη2 = 0.63, Figure 3A]. WCWI and LCWI resulted in a significantly lower T_skin_ than the CON condition from 9 min after the start of the recovery period to the end of the second Yo-Yo. Meanwhile, the T_skin_ with WCWI decreased significantly compared with that of the LCWI intervention from 9 min after the start of the recovery period to the middle of the second Yo-Yo. For T_core_, there was no significant interaction [F (46, 460) = 0.71, *p* > 0.05, Pη2 = 0.07, Figure 3B], but there were significant main effects for the time [F (23) = 70.81, *p* < 0.05, Pη2 = 0.88]. For T_skin_, there was a significant interaction effect [F (46, 460) = 17.18, *p* < 0.05, Pη2 = 0.63, Figure 3A]. WCWI and LCWI resulted in a significantly lower T_skin_ than the CON condition from 9 min after the start of the recovery period to the end of the second Yo-Yo. Meanwhile, the T_skin_ with WCWI decreased significantly compared with that of the LCWI intervention from 9 min after the start of the recovery period to the middle of the second Yo-Yo. For T_core_, there was no significant interaction [F (46, 460) = 0.71, *p* > 0.05, Pη2 = 0.07, Figure 3B], but there was a significant main effect for the time [F (23) = 70.81, *p* < 0.05, Pη2 = 0.88].

**FIGURE 4:**
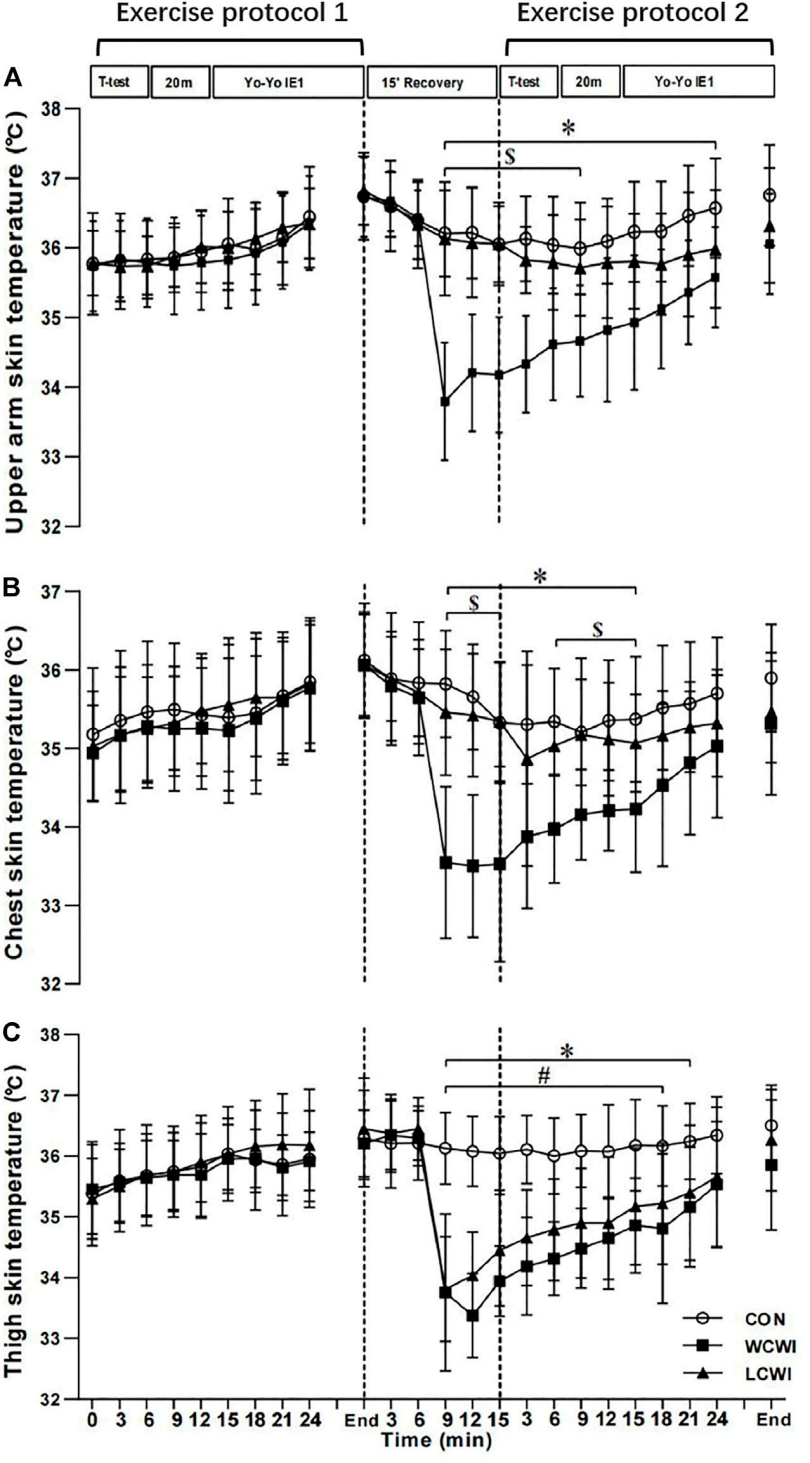
Upper arm skin temperature **(A)**, chest skin temperature **(B)** and thigh skin temperature **(C)** changes at different time points during exercise protocol for control (CON), lower-limb cold-water immersion condition (LCWI) and whole-body cold-water immersion condition (WCWI) conditions. *t*-test, agility *t*-test. 20 m, 20 m sprint test. Yo-Yo IE1, Yo-Yo intermittent endurance test level 1. *, CON vs. WCWI. #, CON vs. LCWI. $, WCWI vs. LCWI. The values are shown as mean ± SD. *p* < 0.05 was considered statistically significant.

For T_arm_, there was a significant interaction [F (46, 460) = 12.61, *p* < 0.05, Pη2 = 0.56, Figure 4A]. WCWI resulted in a significantly lower T_arm_ than the CON condition from 9 min after the start of the recovery period to the middle of the second Yo-Yo. Moreover, the T_arm_ with WCWI was significantly lower than that with LCWI from 9 min after the start of the recovery period to the start of the second 20M-ST. For T_chest_, there was a significant interaction [F (46, 460) = 6.45, *p* < 0.05, Pη2 = 039, Figure 4B]. WCWI resulted in a significantly lower T_chest_ than the CON and LCWI conditions from 9 min after the start of the recovery period to the end of the second 20M-ST. For T_thigh_, there was a significant interaction [F (46, 460) = 11.96, *p* < 0.05, Pη2 = 0.55, Figure 4C]. WCWI and LCWI resulted in a significantly lower T_thigh_ than the CON condition from 9 min after the start of the recovery period to the middle of the second Yo-Yo.

**FIGURE 5:**
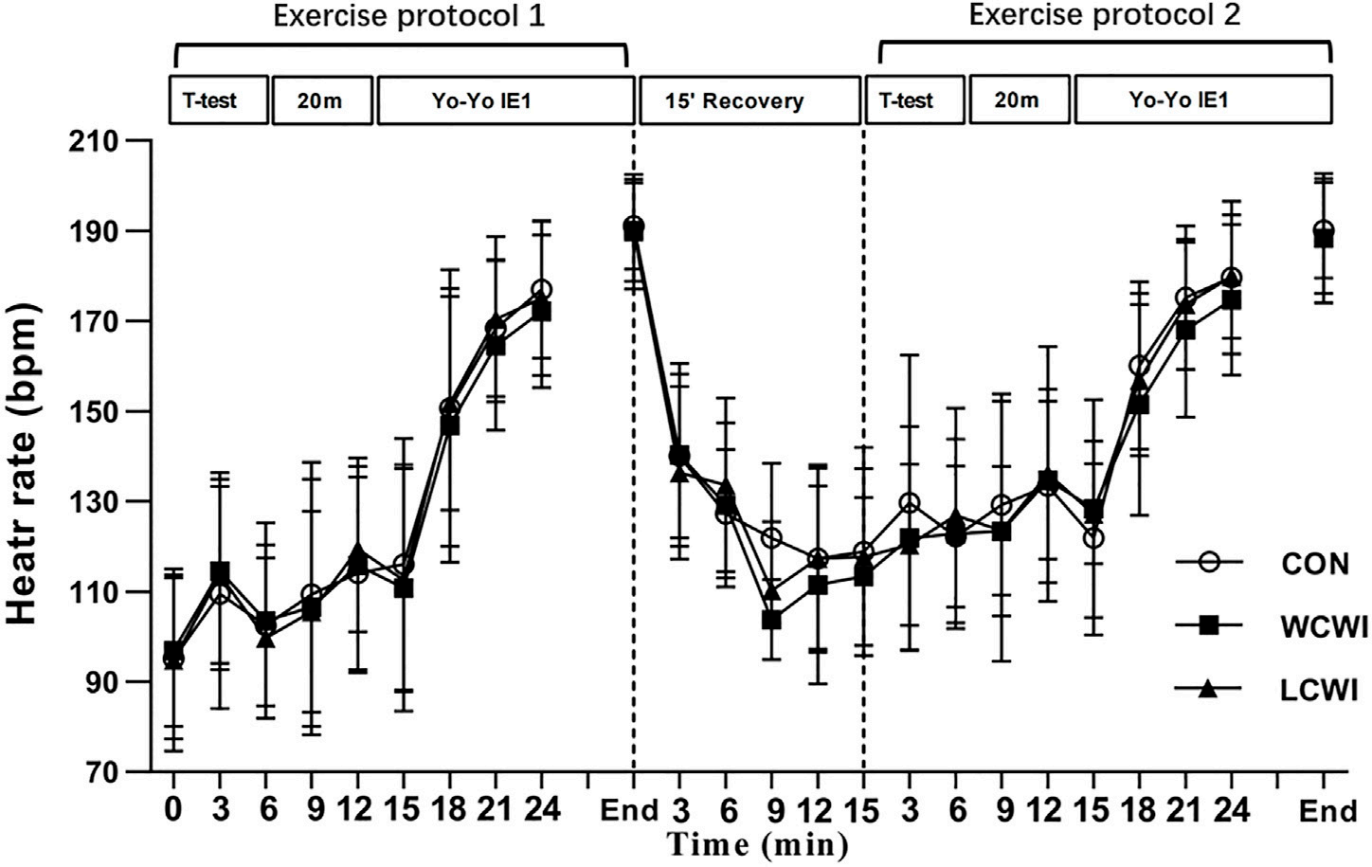
Heart rate changes at different time points for control (CON), lower-limb cold-water immersion condition (LCWI) and whole-body cold-water immersion condition (WCWI) conditions. *t*-test, agility *t*-test. 20 m, 20 m sprint test. Yo-Yo IE1, Yo-Yo intermittent endurance test level 1. The values are showed as mean ± SD.

For HR, there was no significant interaction [F (46, 460) = 0.71, *p* > 0.05, Pη2 = 0.07, Figure 5], but there was a significant main effect for the time [F (23) = 119.46, *p* < 0.05, Pη2 = 0.92].

### 3.4 Perceptual index

The TS values are shown in Table 1. There was a significant interaction for TS [F (22, 220) = 10.58, *p* < 0.05, Pη2 = 0.51; Table 1]. The TS was significantly lower with the WCWI intervention compared with the CON intervention from 1 min of the recovery intervention to after the second 20M-ST. Similarly, the TS was significantly lower with the LCWI intervention compared with the CON intervention at 1, 2, and 3 min during the recovery intervention as well as after the second 20M-ST. Moreover, from 1 min after the start of the recovery intervention to after the second *t*-test, the TS was significantly lower for the WCWI condition compared with the LCWI condition.

**TABLE 1:** Thermal sensation (TS) during exercise protocol.

Time	Time point	CON	WCWI	LCWI
Exercise Protocol 1	Agility *t*-test	5.45 ± 0.82	5.55 ± 1.04	5.27 ± 1.91
	20M-ST	5.91 ± 0.94	6.36 ± 1.12	6.18 ± 1.08
	Pre-Yo-Yo	5.55 ± 0.93	5.36 ± 1.29	5.27 ± 1.01
	Post-Yo-Yo	7.73 ± 0.47	7.73 ± 0.47	7.91 ± 0.30
Recovery period (min)	7th	5.27 ± 1.01	1.73 ± 0.91	2.64 ± 1.29
	8th	5.27 ± 1.01	1.91 ± 0.30*^#^	2.91 ± 0.70*
	9th	4.82 ± 0.87	2.18 ± 0.60*^#^	3.09 ± 0.70*
	10th	4.55 ± 0.93	2.73 ± 0.79*^#^	3.45 ± 0.69*
Exercise Protocol 2	Agility *t*-test	6.18 ± 0.87	4.82 ± 1.78*^#^	5.18 ± 1.08
	20M-ST	6.91 ± 0.70	5.73 ± 1.42*	6.00 ± 1.27*
	Pre-Yo-Yo	5.73 ± 1.01	5.18 ± 1.54	5.45 ± 1.21
	Post-Yo-Yo	7.82 ± 0.41	7.64 ± 0.51	7.82 ± 0.41

CON, control group; WCWI, whole-body cold-water immersion condition; LCWIL, lower-limb cold-water immersion condition; Yo-Yo, Yo-Yo intermittent endurance test level-1; 20M-ST, 20 m sprint test. *: significantly different with CON. #: WCWI, vs; LCWI, The values are shown as mean ± SD. *p* < 0.05 was considered statistically significant.

**FIGURE 6:**
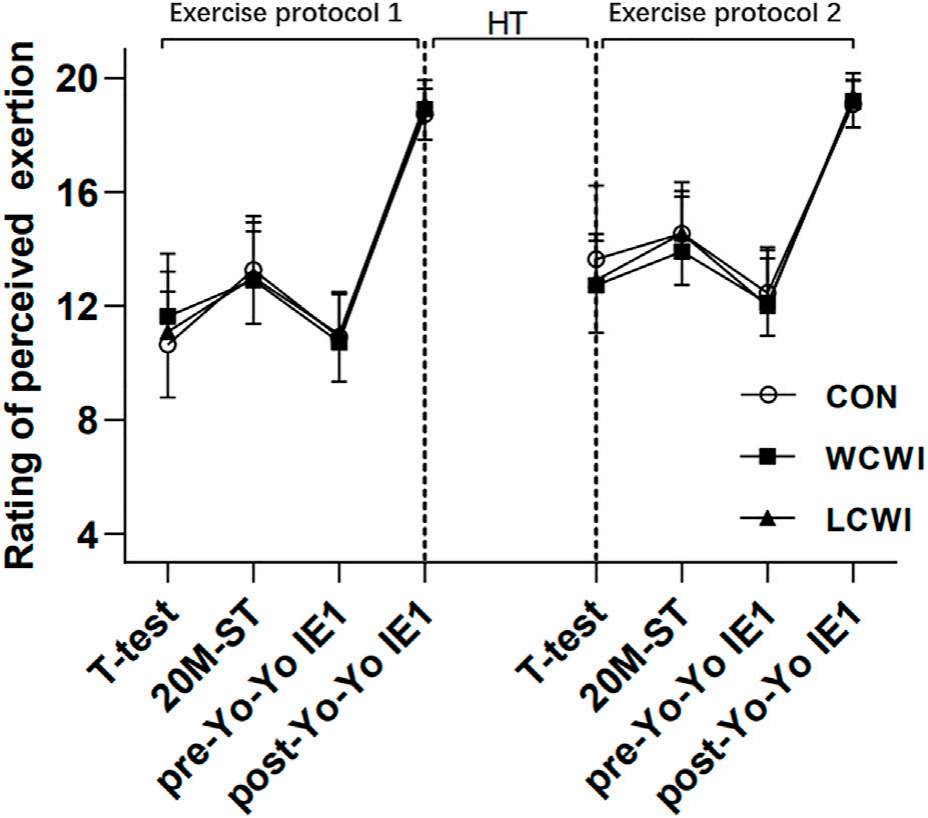
Change in rating of perceived exertion for control (CON), lower-limb cold-water immersion condition (LCWI) and whole-body cold-water immersion condition (WCWI) conditions. *t*-test, agility *t*-test. 20 m, 20 m sprint test. Yo-Yo IE1, Yo-Yo intermittent endurance test level 1. RP, recovery period.

For the RPE, a 3 × 8 mixed analyses of variance revealed that there was no significant interaction [F (14, 140) = 1.36, *p* > 0.05, Pη2 = 0.12, Figure 6], but there was a significant main effect for the time [F (7) = 156.00, *p* < 0.05, Pη2 = 0.94].

## 4 Discussion

In this study, we investigated the effects of 3-min WCWI vs. LCWI during a 15-min recovery period on the subsequent exercise performance in the heat as well as their physiological and perceptual parameters. Our findings demonstrated that compared with the CON condition, LCWI between the two exercise protocols was effective at inhibiting the impairment of the subsequent performance in the *t*-test and 20M-ST in the heat as well as decreasing the T_thigh_, T_skin_, and TS. In addition, the use of WCWI between the two exercise protocols resulted in significant attenuation of the impairment of the subsequent performance observed with the CON condition on the *t*-test, 20M-ST, and Yo-Yo as well as decreased the T_arm_, T_chest_, T_thigh_, T_skin_, and TS values.

As it is well known, the ability of team-sports players to produce varied high-speed actions can affect match performance. High-speed actions during team-sports competitions can be categorized into actions requiring acceleration, maximal speed, or agility (Little and Williams, 2005; Arslan et al., 2021). It has been reported that the 20M-ST can evaluate acceleration and maximum speed capacities (Bangsbo, 1994), while the *t*-test can assess agility. In the present study, we found that the *t*-test and 20M-ST durations were significantly longer during exercise protocol two in the CON condition at intermission. These results are supported by previous studies showing that high-speed actions, which are related to the neuromuscular performance, decrease at the later stages of a match or after a specific field test (Rowsell et al., 2009; Magalhaes et al., 2010; Lovell et al., 2013). Moreover, a team sport like soccer is a physically demanding intermittent sport that stresses both the anaerobic and aerobic energy systems, and the intermittent-exercise performance of athletes is also related to their match performance (Ranchordas et al., 2018). By employing the Yo-Yo test, we observed that the intermittent-exercise performance of the athletes was decreased during exercise protocol two in the CON condition. These results are consistent with those of Chaen et al. (2019), who showed that the intermittent-sprint performance of soccer players was decreased during the second half of the match in the heat. Thus, herein, we demonstrated that high-speed actions and intermittent performance, which often dictate winning or losing a match in many team sports, were decreased after a passive recovery period and that the convenient and practical cooling strategies investigated in this study attenuated the decline in these high-speed actions and intermittent performance of our athletes in the heat.

Some previous studies have explored the effects of post-exercise WCWI on the recovery of high-speed sprint performance. For instance, Bouzid et al. (2018) have reported that 10-min WCWI at 10°C (+1°C) induced the fastest recovery of 20M-ST performance after 24 h, but it failed to affect the physical performance of the athletes immediately after the intervention. Leeder et al. (2019) found similar effects as Bouzid et al. (2018) and observed that 14-min WCWI at 14°C did not affect the 15-m sprint performance of athletes 1 h after the intervention. However, the duration of CWI that was employed in these previous studies (10–15 min) was longer compared to that in our study and may induce overcooling, impair the contractile apparatus of the cooled muscles, and decrease the high-speed actions of the athletes immediately following the intervention (Roberts et al., 2014; Roberts et al., 2015). By employing a 3 min CWI at 15°C within a 15-min recovery period, the present study showed that WCWI as well as LCWI inhibited the decreased performance of the *t*-test and 20M-ST during exercise protocol 2. These results suggest that 3-min WCWI or LCWI can be applied at half-time periods during team sport matches to attenuate the impairment of the subsequent initial, brief, and all-out-effort speed actions.

Furthermore, we found that WCWI inhibited the decrease in the distance of the Yo-Yo test observed in the passive resting condition, and these results indicate that 3-min WCWI attenuates the impairment of intermittent-running performance during exercise protocol 2. The ergogenic effects observed in the present study agree with those of Egaña et al. (2019), who reported significant benefits on intermittent-cycling performance in the second half following a 2.5-min WCWI period within a 15-min recovery period between two equal 40-min “halves” under normothermic conditions. However, this group also reported that 5-min CWI did not enhance the subsequent 4-km cycling time under similar conditions, suggesting that WCWI might be a more efficient method to enhance exhaustive intermittent efforts than time trial efforts, at least under normothermic conditions (Egaña et al., 2021).

Until now, many studies have employed LCWI as a precooling method, demonstrating a positive effect on subsequent intermittent-exercise performance (Castle et al., 2006; Castle et al.,2011; Minett et al., 2011). In addition, Dunne et al. (2013) observed that a 15-min LCWI at either 8°C or 15°C between repeated exhaustive exercise bouts significantly enhanced the post-immersion running time to failure of athletes under normothermic conditions. These positive effects of LCWI may also attenuate the impairment of the subsequent intermittent-sprint performance. We found that LCWI between the two exercise protocols failed to affect the subsequent intermittent sprint performance in the heat employing 3 min LCWI at 15°C. As the findings by Dunne et al. (2013) suggest that a water temperature of 8°C was marginally superior than a water temperature of 15°C for subsequent high-intensity continuous-running performance, 3-min LCWI between the two exercise protocols at 8°C may improve the subsequent intermittent performance in the heat. Therefore, future studies should explore the effects of lower water temperatures on the performance of athletes in the heat subjected to LCWI at intermission.

Previous studies have indicated that the T_skin_ is reduced *via* cooling due to WCWI either before or after exercise. For example, 30-min WCWI at 22°C before exercise reduced the T_skin_ during constant cycling in the heat (Choo et al., 2019). Employing the same WCWI strategy before cycling, Moss et al. (2021) also observed a reduction of the T_skin_ in the heat after the intervention. In addition, WCWI (11.5°C for 60 s repeated three times) after cycling in the heat decreased the T_skin_, and the reduction was maintained for more than 30 min (Halson et al., 2008). In these previous studies, the reduction of the T_skin_ was maintained for more than 30 min. However, the effects of WCWI during the recovery period on the T_skin_ had not been examined in the heat. Comparable to previous studies, the current study revealed that WCWI during a recovery period decreased the T_arm_, T_chest_, and T_thigh_, resulting in a reduction of the T_skin_ throughout the exercise period. Moreover, Hayashi et al. (2004) have reported that 5-min LCWI at 20°C within a recovery period decreased the T_thigh_ and T_skin_ during the subsequent moderate exercise in the heat. Similar to Hayashi et al. (2004), we found that LCWI during a recovery period had a beneficial effect on the T_thigh_, thus inducing a reduction in the T_skin_. However, in contrast to a previous study (Hayashi et al., 2004), LCWI in the present study did not induce an effect on the T_chest_. These inconsistent results of the T_chest_ *via* LCWI during a recovery period are possibly due to differences in the ambient temperature or durations of LCWI.

Comparable to previous studies (Halson et al., 2008; Vaile et al., 2008; McCarthy et al., 2016), the present study demonstrated that TS was decreased after the WCWI and LCWI interventions. Furthermore, similar to the findings of Egaña et al. (2019), WCWI and LCWI in the present study enhanced the subsequent motor performance but failed to affect the RPE, and these results showed that the perceived effort did not increase with the output results. Our data indicate that these two cooling methods during a recovery period successfully inhibited the enhancement of the RPE during the subsequent exercise in the heat. Taylor et al. (2020) have reported that effective cooling during exercise can reduce the skin temperature, resulting in a decreased thermal perception and relieving the thermal strain sent from the peripheral thermoreceptors to the hypothalamus. Therefore, the present study demonstrated that WCWI and LCWI have beneficial positive effects on the TS and RPE, which are related to the decrease in skin temperature. Moreover, the inhibition of perceived strain (e.g., TS, RPE) can suppress the selective decrease in exercise intensity and then induce the improvement of motor performance (Taylor et al., 2020). Therefore, the ergogenic effects of WCWI and LCWI between the two exercise protocols may be due to the decrease in skin temperature and perceived strain. Furthermore, compared with LCWI, WCWI caused a greater decrease in the T_skin_ that lasted for longer, thus explaining why WCWI showed larger ergogenic effects than LCWI.

It has been reported that increases in the T_core_ can result in a reduced exercise performance (Nybo et al., 2014) and that the ergogenic effects of CWI are linked to a decrease in the T_core_ (Versey et al., 2013). A 2.5-min WCWI at 8°C during a 15-min recovery period has been demonstrated to reduce the T_core_ during the subsequent intermittent-cycling performance under normothermic conditions. In addition, Hayashi et al. (2004) described that 5-min LCWI at 20°C within a recovery period decreased the T_core_ during subsequent moderate exercise in the heat. The present study showed that WCWI as well as LCWI failed to decrease the T_core_. These inconsistent results may be due to the different intervention strategies or experimental ambient temperatures. Additionally, we also found that WCWI and LCWI did not affect the HR. These findings suggest that a relatively short cryotherapy time would be unlikely to cool the body’s core tissues and reduce cardiovascular strain in the heat.

It should be acknowledged that this study had some limitations. Although the participants in the present study were all collegiate soccer players, these results are probably not specific to soccer players and might be observed in other sports as well, provided that the same athletic circumstances are present. In addition, in the present study, the mean WGBT was 39°C, which is considered a very high/extreme temperature, and can cause a heat-induced illness. This might limit the practicality of the current results. Moreover, we did not measure the technical aspects of actual match play. Future studies should investigate the effects of WCWI and LCWI on the technical aspects of team sports as well as actual match play. Finally, we did not measure the muscle temperatures, and these indexes should also be explored in future studies.

## 5 Conclusion

Compared with a passive recovery, WCWI for 3 min during a 15-min recovery period attenuated the impairment of agility, sprint, and intermittent endurance performance of athletes during exercise protocol 2, but LCWI only ameliorated the reduction of agility and sprint performance. Furthermore, the ergogenic effects of WCWI and LCWI in the heat were due, at least in part, to a decrease of the T_skin_ and improvement of perceived strain. We suggest that 3 min WCWI and LCWI at 15°C within a 15 min recovery period could be used to enhance performance in the second half of a team-sport match in the hyperthermia.

## Data Availability

The raw data supporting the conclusion of this article will be made available by the authors, without undue reservation.
